# Omega-3 Long-Chain Polyunsaturated Fatty Acids and Preeclampsia: Trials Say “No,” but Is It the Final Word?

**DOI:** 10.3390/nu9121364

**Published:** 2017-12-15

**Authors:** Denis I. Burchakov, Irina V. Kuznetsova, Yuliya B. Uspenskaya

**Affiliations:** Clinic of Obstetrics and Gynecology n.a. V.F. Snegirev, Department of Obstetrics and Gynecology No. 1, I.M. Sechenov First Moscow State Medical University of the Ministry of Health of the Russian Federation (Sechenov University), 8-2 Trubetskaya st., 119991 Moscow, Russia; ms.smith.ivk@gmail.com (I.V.K.); jusp@mail.ru (Y.B.U.)

**Keywords:** pregnancy, preeclampsia, docosohexaenoic acid, supplementation

## Abstract

Preeclampsia is a dangerous disorder of pregnancy, defined as hypertension with proteinuria. Its nature remains elusive, and measures of prevention and treatment are limited. Observational studies have suggested that preeclampsia is associated with low intake of omega-3 long-chain polyunsaturated fatty acids (LCPUFA). In recent decades, researchers studied LCPUFA supplementation as a measure to prevent preeclampsia. Most of these trials and later systematic reviews yielded negative results. However, these trials had several important limitations associated with heterogeneity and other issues. Recent research suggests that preeclampsia trials should take into consideration the gender of the fetus (and thus sexual dimorphism of placenta), the positive effect of smoking on preeclampsia prevalence, and the possibility that high doses of LCPUFA mid-term or later may promote the disorder instead of keeping it at bay. In this review, we discuss these issues and future prospects for LCPUFA in preeclampsia research.

## 1. Introduction

Hypertensive disorders in pregnancy are increasingly common and lead to substantial maternal, fetal, and neonatal morbidity and mortality. Methods to diagnose and treat these disorders evolve over time, but in many cases, only immediate cesarean section can save a mother and child from quickly developing systemic malfunction. According to an American College of Obstetricians and Gynecologists (ACOG) Task Force document, there are four clinical categories of hypertension in pregnancy: chronic hypertension, preeclampsia–eclampsia, preeclampsia superimposed upon chronic hypertension, and transient hypertension [1]. Among these, preeclampsia is undeniably the most dangerous, leading to 10–15% maternal deaths worldwide [2]. 

Preeclampsia is a protean syndrome and is better described as a spectrum, not a single well-defined entity. Since it affects virtually all systems, its clinical presentation can vary wildly. The diagnosis of preeclampsia, in addition to the de novo appearance of both hypertension and proteinuria after mid pregnancy, now includes the appearance of high blood pressure in association with thrombocytopenia, impaired liver function, renal insufficiency, pulmonary edema, or cerebral or visual disturbances when these latter findings are new in onset [3]. Preeclampsia occurs more frequently in nulliparous women and becomes more frequent closer to term. When preeclampsia manifests early, there is high probability that it is superimposed with essential hypertension or renal disorder. Contemporary studies often utilize the terms “mild” and “severe” preeclampsia. Severe disease is usually defined by systolic and diastolic blood pressure levels above 160 and 110 mm Hg, nephrotic range proteinuria, neurological symptoms, thrombocytopenia, and/or hepatocellular disruption. However, devastating eclamptic convulsions may suddenly appear even in “mild” cases. Therefore, rather than defining the syndrome as mild or severe, it would be more accurate to describe it as preeclampsia with or without severe features [1]. 

Despite researchers’ best efforts the nature of preeclampsia remains elusive and methods of prevention and management are limited. Data from observational studies suggest that preeclamptic women have lower levels of omega-3 long chain polyunsaturated fatty acids (LCPUFA) [4,5]. This motivated researchers to test whether omega-3 LCPUFA supplementation could decrease the risk of preeclampsia in clinical trials [6,7,8,9,10,11,12,13,14]. Most of these studies and later systematic reviews produced negative results. Why did this happen? Basically, there are two scenarios: either observational studies were wrong in the first place, or clinical trials could not detect the effect due its limited size or due to their design limitations. Below, we discuss some evidence concerning LCPUFA and preeclampsia and then describe potential factors that could limit trial sensitivity.

## 2. Biological Effects of LCPUFA

Omega-3 LCPUFA (also known as *n*-3 fatty acids) have a double bond at the third carbon atom from the methyl end of the carbon chain. Some of them are essential, particularly alpha-linoleic acid (ALA), eicosopentaenoic acid (EPA), and docosohexaenoic acid (DHA). The latter two can be obtained by conversion from ALA, but in humans, this conversion seems to occur at a low level [15]. Major dietary sources of omega-3 LCPUFA are fish and seafood. Consequently, their levels vary worldwide, depending on local dietary customs. Omega-3 functions are best understood in the context of their interaction with omega-6 LCPUFA, derivatives of linoleic acid (LA), arachidonic acid (AA) being most significant among them. For the sake of brevity, we will further refer to these two families of LCPUFA as simply omega-3 and omega-6, indicating the specific acid when appropriate.

LCPUFA are present in all human tissues and required for synthesis of structural and functional lipids. They may also play a proprietary structural role, which have functional implications. For example, serotonin receptor depends on cell membrane fluidity, which in turn depends on the balance between cholesterol and DHA [16]. Since the serotonin receptor transverses the cell membrane seven times, its ability to bind serotonin decreases when the membrane is less fluid due to relative lack of DHA [17]. This mechanism is of primary clinical importance in mental health, and indeed omega-3 LCPUFA are now under study in this field. Since serotonin neurons are specified and matured during the first month of gestation in primates, this mechanism also seems important for neurodevelopment of the embryo [18].

Omega-3 and omega-6 LCPUFA compete for enzymes and are converted to various bioactive compounds. Omega-6 derivatives promote inflammation, while omega-3 derived agents (such as resolvins and protectins) exert opposing effect [19]. Therefore, a balance of LCPUFA is important for normal inflammatory response. They are also involved in maintenance of the balance between generation of reactive oxygen and nitrogen species and antioxidant defense. Both inflammation and oxidative stress are involved in critical reproduction events: ovulation and implantation. Excessive inflammatory and/or oxidative stress may disrupt the process, potentially leading to pregnancy disorders. Leghi and Muhlhaussler provide a broad overview of these topics, discussing relevant research in humans and animal models [20]. 

## 3. LCPUFA Over the Course of Pregnancy

The overall level of maternal fatty acids undergoes increase during pregnancy to satisfy the needs of the growing fetus. However, when researchers evaluated the amounts of individual fatty acids, they saw that despite initial mobilization, DHA status declines after 18 weeks of gestation. Meanwhile, the DHA deficiency index (a measure of functional DHA status [21]) in maternal blood increases, resulting in sub-optimal neonatal DHA status [22]. These changes are most notable in the third trimester, when the fetus rapidly accumulates LCPUFA in brain and retina. Supplementation of 400 mg DHA, as assessed by double-blind randomized controlled trial, significantly limits but does not prevent this decline [23]. 

How does the presence or absence of fish in diet affect these changes? Kawabata et al. studied LCPUFA levels in a fish-eating population of Japanese women. Median maternal DHA at week 27 was 6.92% (of total fatty acids) and decreased significantly toward delivery, thus reflecting the transfer to the fetus [24]. But when maternal DHA status is persistently low, this transfer is compromised. In one study, researchers monitored women from South India who consumed no more than 11.2 mg DHA per day (3% of locally recommended 300 mg). Their erythrocyte membrane DHA concentration was low and did not change significantly across trimesters. This steady level may be maintained by conversion from ALA and also by diminishing of DHA transport to the fetus [25]. 

In another study, within a low DHA intake setting, term mothers and preterm infants exhibited significantly better DHA levels compared to term infants [26]. Therefore, if maternal DHA stores are low, only limited amount of it can be transferred to fetus during late pregnancy, thus limiting fetal development. These findings parallel recent studies on magnesium status during pregnancy. In mice fed with moderately magnesium-deficient diet, mothers have lower plasma magnesium levels, while fetuses did not differ from controls in this regard. However, when fed a severely magnesium-depleted diet, mothers actually have higher magnesium levels [27]. It is possible that at a certain point, the maternal organism partially deprives the fetus of LCPUFA in order to complete the pregnancy without suffering substantial damage.

## 4. Preeclampsia, Placenta, and LCPUFA

Preeclampsia manifests at mid-term or later, but according to contemporary theories, the disease has two stages: abnormal placentation and maternal reaction to it. 

### 4.1. Normal and Abnormal Placentation

During normal development of placenta fetal cytotrophoblasts invade maternal spiral arteries. Consequently, these small-caliber resistance vessels transform into high-caliber capacitance vessels. They lose their endothelial lining and muscoloelastic tissue. These changes in architecture allow them to contain increased blood flow. In preeclampsia cytotrophoblasts invasion is shallow. They remain at distance from the arteries, and even if they invade some, they do not produce sufficient transformation. Thus, when placental blood flow increases in response to increased fetal needs, abnormal placentation results in ischemia [28]. The causes of abnormal placentation are unclear. 

Fatty acids in general, and LCPUFA in particular, play numerous roles at this stage of the pregnancy. During implantation, they are required as structural components and functional regulators for normal a growth process [29]. AA and its prostaglandin derivatives are important as mediators of vascular permeability and cytotrophoblast invasion. However, excessive action of prostaglandins may result in implantation failure [30]. Therefore, abnormal omega-3/omega-6 ratio may support excessive inflammatory and oxidative events, that hamper or disrupt embryo development and placentation. Later on, during the first trimester, LCPUFA stimulate the expression of various factors, that help trophoblasts in their angiogenic mission [31,32]. 

### 4.2. Maternal Reaction and Manifestation of Preeclampsia

Maternal reaction to abnormal placentation is not completely understood. Most researchers agree that endothelial dysfunction and oxidative stress are key components of systemic reaction and the development of clinical symptoms. Endothelial dysfunction occurs due to the imbalance of numerous circulating factors. Among these are fibronectin, endothelin, thrombomodulin, endoglin, and others [33,34,35,36,37]. Recent studies also indicate that women with preeclampsia have elevated levels of sFlt-1 (soluble fms like tyrosine kinase-1). This molecule is a protein derived from a splice variant of the vascular endothelial growth factor (VEGF) receptor Flt-1 mRNA. It lacks the transmembrane and cytoplasmic domain of the membrane-bound receptor [38,39]. Placenta secretes sFlt-1 and releases it into the maternal bloodstream, where it can disrupt endothelial function, provoking hypertension, proteinuria, and other systemic manifestations of preeclampsia. It is not clear, what induces the release of sFlt-1. However, according to Kulkarni et al., a low level of DHA may be one of the reasons [40]. The authors compared proportions of LCPUFA and sFlt-1 in 69 women with preeclampsia and 40 normotensive controls. Compared to controls, preeclamptic women had significantly lower placental DHA (3.32% vs. 2.86%, *p* = 0.004) and omega-3/omega-6 ratios. They also had higher plasma levels of sFlt-1, which were negatively associated with DHA (*p* = 0.008). 

Oxidative stress in placenta occurs naturally, when invading cytotrophoblasts encounter a steep positive oxygen tension gradient. This gradient might regulate cytotrophoblast proliferation and differentiation [41]. LCPUFA play dual role in this process. Omega-3 fatty acids and particularly DHA reduce placental oxidative stress and increase the levels of resolvins and protectins that also help to keep oxidation at bay. However, the free radicals peroxidize LCPUFA produce toxic molecules [42]. This ambivalence may partially explain failure of LCPUFA to prevent preeclampsia in clinical trials. Supplementation contributes simultaneously to beneficial and harmful effects, thus negating itself. 

An interesting avenue of research here is LCPUFA distribution in placenta. Rani et al. assessed placentas of (1) normotensive women; (2) women with preeclampsia and term birth and (3) women with preeclampsia and preterm birth. The latter had lower proportions of DHA and other omega-3 acids in central maternal and central fetal regions of placenta [43]. Preeclampsia with preterm birth in clinic usually means, that maternal condition was dangerous enough to justify the urgent cesarean section. In these patients’ endothelial dysfunction, oxidative stress and other factors behind the abnormal reaction are especially strong. It is unclear whether depletion of DHA precedes or follows preeclampsia manifestation. Hopefully, future studies will help establish causative links.

## 5. Studies Concerning Association between LCPUFA and Preeclampsia

Any researcher or practitioner willing to learn about effects of omega-3 on pregnancy-induced hypertension and preeclampsia can scan recent systematic reviews and meta-analyses. Among these are Cochrane review [44], meta-analysis of six RCTs concerning low-risk pregnancies [45], a meta-analysis of four RCTs concerning high risk pregnancies [46], a systematic review including five relevant studies [47], and another recent review [48]. Their conclusion is uniform—clinical trials show no significant effect. This may be the case either because LCPUFA are indeed ineffective or because the design of the analyzed trials does not take in account certain factors. In this section, we discuss possible confounders, starting from basic heterogeneity issues and then moving to smoking and gender of the fetus. We mostly consider randomized controlled clinical trials with omega-3 fatty acids supplements included in aforementioned reviews. Table 1 presents a brief summary of these studies. With it, we do not seek to present the results of the studies—which are best described in a meta-analysis—but rather to show great variety in study design.

This table requires a few comments. First, in all included studies, the authors used incidence of preeclampsia (hypertension plus proteinuria) as an outcome. Criteria vary, especially in early works. Sometimes there were other outcomes, but they are beyond the scope of this review. Second, we adjusted patient numbers for exclusion, dropout, and relevance. For example, D’Almeida enrolled 150 patients, but 50 of them went to a group receiving magnesium. We chose to skip this group. Third, we also included two studies with LA, an omega-6 fatty acid. Recent study showed that in rats, high LA intake does not decrease DHA synthesis, while low LA intake does [49]. Finally, reports of gestational age and treatment duration vary significantly in measures. We adhered to data in the original papers. 

### 5.1. Basic Issues

Reviewers routinely assess trial heterogeneity. The omega-3 and preeclampsia trials differ significantly in type of omega-3 supplementation, doses and forms. While now there seems to be more experimental evidence for DHA in preeclampsia, most studies utilized either pure EPA supplementations or EPA and DHA with higher dose of the former. This trend continues in omega-3 research: a recent triple blind randomized controlled trial studied effects of 180 mg EPA plus 120 mg DHA started at 20 week of pregnancy [50]. Authors observed some effect on birth weight, but it wasn’t statistically significant. In the context of the findings outlined above this is not surprising: EPA:DHA ratio of 3:2 does not seem to be effective. However, there are data showing that DHA is a better modulator of inflammation markers [51]. Therefore, there is still a possibility that other ratios may produce significant effect.

Selection of an optimal dose is also an open question. When symptoms of preeclampsia develop (during the second stage of the disease), excessive amounts of omega-3 may promote oxidative stress or inflammation instead of curtailing them. In an interesting study, Olafsdottir et al. evaluated risk of pregnancy-induced hypertension (PIH) and preeclampsia in a population with high consumption of cod liver oil rich in LCPUFA [52]. Authors divided LCPUFA consumption assessed between 11 and 15 weeks of gestation into centiles and discovered a U-shaped curve for the risk of hypertensive disorders. Odds ratios were lowest for 100–900 mg omega-3 consumption per day. Also, according to Clausen et al., high consumption of PUFA is associated with increased risk of preeclampsia [53]. Combined with clear failures of high-dose supplementation in clinical trials this seems a valid reason to reduce study dosages. The next possible step is individual dosages. In the DOMInO trial, DHA capsules explained only 21% of the variation in DHA abundance in cord blood plasma phospholipids at delivery [54]. However, no other lifestyle or clinical factor could explain >2% of the variation. Reflecting on this finding, Leghi et al. suggest that there are probably significant inter-individual differences in maternal, placental, and/or fetal metabolism. Thus, best dose for best outcome may vary [20]. 

Another possible limiting factor is a timing of supplementation. For example, in 2012, Zhou et al. presented the results of DOMInO, a double-blind multicenter randomized controlled trial. Researchers randomized 2399 pregnant women of less than 21week gestation to receive either DHA-enriched fish-oil (800 mg/day) or vegetable oil. The study was adequately powered with 1197 and 1202 women included in final comparison between two groups. Still, there were no differences in adjusted or unadjusted risk of preeclampsia or pregnancy-induced hypertension [14]. While these results seem conclusive, the authors do note that they cover only second half of the pregnancy, since median gestational age at the baseline was 19 weeks. Compared to other studies, this is relatively early, since most of them have mean gestational age in range from 21 to 35 weeks. We speculate that early studies perceived omega-3 as an agent with anti-hypertensive effects, thus justifying mid-term or later administration. However, even if they do exert such effect, it is too small to control hypertension. Also, according to contemporary model of preeclampsia outlined above, this disorder originates from early placentation problems [3,28,39]. The mid-term rise of inflammation and disruption of microcirculation are the consequences of these earlier problems. It is also possible, that omega-3 supplementation from mid-term does not work simply because it is too late for it to exert significant effect. This may be the reason behind findings of Popeski et al. Authors studied Canadian Inuit population. They saw, that women from communities with lower marine food consumption are 2.6 times more likely to be hypertensive during pregnancy (*p* = 0.007) [55]. To the best of our knowledge there are no studies evaluating influence of omega-3 supplementation given before conception or in the first weeks of pregnancy on later outcomes. 

### 5.2. Gender of the Fetus

O’Tierney-Ginn et al. studied 40 maternal-neonatal pairs of uncomplicated term pregnancies. They analyzed maternal weight gain before pregnancy and across trimesters, placental weight and size, neonatal outcomes and the blood levels of fatty acids. Umbilical venous plasma DHA, total omega-3 LCPUFA and the placental thickness were negatively associated with first trimester weight gain. Strikingly, these associations were only observed in male neonates [56]. Thus, high-fat diet, leading to quick weigh gain may compromise placenta development. Since the male fetus grows quicker compared to the female and also requires more fatty acids for active steroidogenesis, this deficit persists. In the perinatal period, it again manifests as low DHA levels, hampering further development. 

These findings reflect an interesting and relatively new problem—sexual dimorphism of the placenta. Gender differences exist in the placenta on many levels: gene and protein expression, immune function, and epigenetic modifications [57]. These differences affect placental response to environmental stressors. Kalisch-Smith et al. provide an extensive overview of this phenomenon and suggest, that dimorphic responses can occur during two critical windows. First is the periconceptional period, which is more dangerous to female embryos, resulting in more males produced at birth. Second is mid-to-late gestation, when fetal nutrient demands increase rapidly, placing male fetuses at risk [58]. Therefore, gender is a significant confounder, and pooling male and female fetuses may limit trial sensitivity. It may also limit our understanding long-term health outcomes. The perception of placenta as an asexual organ is now clearly outdated. 

The existing reports concerning fetal gender influence on PIH and preeclampsia are not fully consistent. A recent survey indicates that female fetuses are at higher risk [59]. This may seem as incompatible with the aforementioned concept of two critical windows, but if one considers preeclampsia as a consequence of problematic implantation, it becomes plausible. Nevertheless, current reports are limited by the lack of universal study framework. Verburg et al. proposed to utilize a so-called fetus-at-risk (FAR) approach, as they did in their study. This approach is accepted and used in stillbirth studies [60]. It identifies fetuses, not neonates, as the candidates for perinatal events. As in survival analysis it takes into account the remaining fetuses at risk. As gestation proceeds and babies are born preterm, outcomes are analyzed only for the remaining fetuses [61]. 

We also advise against pooling pregnancy-induced hypertension and preeclampsia in one outcome. While these two conditions could develop sequentially, preeclampsia is a far more complicated and dangerous condition. This distinction should be also acknowledged in healthcare databases, since many studies rely on them for raw data. Wietrak et al. demonstrated, that DHA supplementation is associated with the inhibition of placental apoptosis in preeclamptic women [62]. This may not be sufficient to prevent the manifestation of preeclampsia but could help limit its symptoms. To detect this effect, researcher may divide preeclampsia into categories based on presenting symptoms. 

### 5.3. Smoking

Smoking is a detrimental habit, especially for pregnant women. Researchers routinely collect data on the smoking status, since it has been associated with preterm birth and low birth weight [63]. This becomes particularly important when preeclampsia is one of the measured outcomes. 

In one study, 852 pregnant women with a history of preterm delivery between 20 and 36 weeks of gestation were assigned to receive either omega-3 supplementation (1200 mg EPA and 800 mg DHA daily) or matching placebo. Intervention lasted from 16–22 weeks until 36 weeks or delivery, whichever occurred first. All participants received 17alfa-hydroxyprogesterone caproate, a mandatory standard of care. Authors found no difference in the rates of preterm delivery or preeclampsia between groups [64]. Even though results may be blunted by progesterone administration, they can hardly speak in favor of omega-3 supplementation. 

However, Kuper et al. recently performed a secondary analysis of these data by stratifying patients into smokers and nonsmokers. Omega-3 supplementation was associated with a lower risk of spontaneous preterm delivery in smokers (RR 0.56, 95% CI 0.36–0.87) but not in nonsmokers (RR 1.04, 95% CI 0.84–1.29); *p*-value for interaction = 0.013. Therefore, omega-3 may be protective in certain populations. This result is interesting by itself. However, discussing preterm birth further is beyond the scope of this review. The authors also checked for other outcomes, including pregnancy-induced hypertension and preeclampsia as one outcome. There were no notable differences between groups. However, the trial was not designed with this specific effect in mind, hence lacking the power to detect it confidently [65]. Nevertheless, this secondary analysis may become an example for future study designs because smoking affects pregnant women in non-obvious ways. 

According to Alpoim et al., smoker status is associated with a 4.72 decreased chance of preeclampsia (OR 4.72, 95% CI 1.51–14.75) [66]. Another study demonstrated that this protective effect does not rely on nicotine alone because, in snuff users, adjusted odds ratio (OR) of preeclampsia were 1.11 (95% CI 0.97–1.28), while light and heavy cigarette smokers had OR 0.66 (95% CI: 0.61 to 0.71) and OR 0.51 (95% CI: 0.44 to 0.58), respectively [67]. Wei et al. performed a systematic review and meta-analysis of 17 prospective studies, including 62,089 preeclampsia patients among approximately 1.8 million subjects. They also found a significant negative association between smoking during pregnancy and preeclampsia (RR = 0.67, 95% CI: 0.60–0.75) [68]. This effect seems to depend on race. Chang et al. report, that compared to non-smokers reduced odds of PIH were only evident for non-Hispanic white and non-Hispanic American Indian women aged less than 35 years. Older women and non-Hispanic Asian/Pacific Islander women had increased odds of PIH [69].

Mechanisms behind this association are unclear, however it seems that smoking affects maternal sFlt-1 and PlGF concentrations and improve their ratio, at least in normotensive women [70]. In the experimental setting, carbon monoxide and overexpression of heme oxygenase-1 lowered sFlt-1 [71,72]. Another possible pathway is the increase of placental adrenomedullin expression. This small protein has multiple functions, including angiogenic, anti-inflammatory, and antimicrobial effects. Cigarette smoke extract increases the invasiveness of trophoblast cells in vitro and in vivo by enhancing adrenomedullin expression. Co-treating the cultured cells with adrenomedullin inhibitor significantly attenuates this effect [73,74].

While the effect of smoking on preeclampsia is not completely understood, it nonetheless represents a potentially significant confounding factor. It seems to work in a dose-dependent manner, since the effect is stronger in heavy smokers. Therefore, there is a need to assess smoking status of pregnant women in clinical trials with better precision, recording when they started to smoke, whether or not they were smoking around conception, how many cigarettes or other tobacco products they have used, etc. Researchers may also benefit from performing secondary analysis of the data from major clinical trials, now stratified by smoking status. These measures will make omega-3 trials more accurate because according to a recent cross-sectional study, nonsmokers consume almost twice as much PUFA-rich foods compared to smokers (800 g vs. 430 g, *p* < 0.001) and have higher DHA proportions (4.81% vs. 4.13%, *p* < 0.05) [75]. Perhaps during pregnancy, smoking protects against preeclampsia by balancing out DHA deficiency with its own protective effects while still causing harm in other areas.

## 6. Conclusions

Omega-3 long chain polyunsaturated fatty acids play an important role in implantation, placentation, and further development of normal pregnancy. In preeclampsia, their status seems to be compromised, though causative links have not been established. Existing clinical trials do not show a significant benefit of omega-3 supplementation for the prevention of preeclampsia. However, these trials have several important limitations. These are the result of limited understanding of the role of omega-3 fatty acids in preeclampsia rather than poor study design. We suggest a trial of primarily DHA-based supplement in high-risk pregnancies, with intervention starting at confirmation of pregnancy or in periconceptional period. The dose of the supplement should not exceed 900 mg per day. A question arises: if this theoretical omega-3 effect requires so much tinkering to detect, can it be clinically significant? A logical question to ask is whether the theoretical omega-3 effect could be clinically significant if it is so hard to detect. The only way to get a definitive answer is to perform a carefully designed analysis that takes into account all potential confounding factors described above. In our opinion, such analysis has the potential to improve future preeclampsia-eclampsia research. Given the enigmatic and dangerous nature of this syndrome, all clinical opportunities that can shed light on its underlying mechanisms should be pursued.

## Figures and Tables

**Table 1:** Randomized studies of omega-3 supplementation and hypertensive disorders of pregnancy.

Author(-s) and Year	Supplementation Per Day According to Methods	Population and Sample with Adjustments for Exclusion, Dropout and Missing Information Where Possible	Gestational Age at Randomization/Baseline and Treatment Duration	Preeclampsia and Hypertension Criteria According to Methods Section
Moodley and Norman, 1989 [6]	Evening primrose oil 400 mg (LA 73% and 8% GLA) vs. placebo	47 (23 + 24) primigravidae pregnancies, singleton or twins not mentioned	Mean 35 (range 30–36) weeks in main group and mean 34 (range 32–36) weeks in control group Duration: minimum 2 weeks	Study included women with established preeclampsia at BP 140/90 mm Hg or more after 24 h of hospital bed rest. Authors mention proteinuria evaluation, but do not disclose details.
D’Almeida et al., 1992 [7]	Evening primrose oil (GLA 296 mg) plus fish oil (DHA 80 mg and EPA 144 mg) vs. placebo	100 (50 + 50) primigravidae and multigravidae singleton pregnancies, 21% had a history of PIH or other hypertensive disorder	First 4 months of pregnancy Duration: 30 days	Preeclampsia defined as simultaneous occurrence of: (1) hypertension: a rise in SBP greater 30 mm Hg and/or a rise in DBP greater than 15 mm Hg; either one or both, during the course of the pregnancy; (2) proteinuria: protein greater than one determined by test tape; (3) edema: visible fluid accumulation in the ankles and feet; indentation produced by pressure applied by the thumb over the anterior surface of the tibia.
Onwude et al., 1995 [8]	EPA 1620 mg and DHA 1080 g vs. placebo	232 singleton pregnancies, including: −35 + 37 primigravidae with abnormal Doppler at 24 weeks −34 + 34 multigravidae with previous birthweight < 3rd centile −36 + 40 multigravidae with history of proteinuric or nonproteinuric PIH −8 + 8 multigravidae with history of unexplained stillbirth	Mean 24.0 (range 18–32) weeks in main group and 24.4 (range 18–32) weeks in control group. Majority of patients 19–26 weeks. Duration: not reported Women obtained 140 capsules and were supposed to take 9 per day. However, 50% of women in fish oil group and 57% in control group took less than 70% of capsules.	Hypertension: diastolic blood pressure of at least 90 mm Hg on two consecutive occasions at least 4 h apart. Proteinuria: two clean-catch-midstream specimens of urine collected > 4 h apart with 1 g/L albumin or 2+ more on reagent strip.
Bulstra-Ramakers et al., 1995 [9]	Mixture of DHA and EPA, approximately 3000 mg of the latter daily vs. placebo	70 multigravidae with 63 completing the study, including: −24 + 15 with history of PIH and IUGR −1 + 1 with history of IUGR and renal disease −7 + 15 with history of IUGR	12–14 weeks Duration: not reported 78% women in fish oil group and 75% in control group reported, that they usually took 4 capsules per day	Hypertension: an increase in diastolic blood pressure of a t least 25 mm Hg in the course of pregnancy, with a final diastolic pressure >90 mm Hg. Proteinuria: albuminuria >0.5 g/24 h.
Salvig et al., 1996 [10]	EPA 1280 mg and DHA 920 mg vs. olive oil vs. placebo	533 (266 + 136 + 131) singleton pregnancies	30 weeks Duration: until delivery	Hypertension: BP greater than 140/90 mm Hg after rest at two subsequent measurements with a 6 h interval. Proteinuria: (>0.3 g/L).
Herrera et al., 1998 [11]	LA 450 mg and calcium 600 mg vs. placebo	86 (43 + 43) primigravidae with high risk of PE: biopsychosocial risk score above 3, positive roll-over test and mean BP > 85 mm Hg.	28–32 weeks Duration: 4 weeks	Hypertension: acute development of hypertension in a woman whose blood pressure was normal in the early stages of pregnancy and who had persistent elevation of blood pressure to at least 140/90 mm Hg, that must have represented an increase in the diastolic pressure of no less than 20 mmHg determined in at least two occasions six or more hours apart. Proteinuria: 24-h proteinuria (more than 0.3 g/L) in the absence of urinary tract infection.
Olsen et al. [12] “Earl-PIH”	EPA 1300 mg and DHA 900 mg vs. olive oil	350 (167 + 183) singleton pregnancies with history of PIH defined as DBP > 100 mm Hg in earlier pregnancy	Mean 18.4 ± 3.05 weeks in main group and 18.9 ± 3.8 weeks in control group *. Duration: until delivery	Hypertension: one or more recorded measurements of a DBP > 90 mmHg at rest. Proteinuria: urinary measurement of > 1+ in albustix, 0.3 g protein/L, 0.3 g protein/24 h, or 300 mmol protein/L.
Olsen et al. [12] “Twins”	EPA 1300 mg and DHA 900 mg vs. olive oil	553 (274 + 279) twin pregnancies, with nulliparity at 52.5% at both groups. Analysis for preeclampsia included 497 (246 + 251) pregnancies due to missing data on proteinuria	Mean 20.2 ± 3.0 weeks in main group and 20.2 ± 3.04 weeks in control group *.Duration: until delivery	Hypertension as one or more recorded measurements of a DBP > 90 mmHg at rest. Proteinuria as a urinary measurement of > 1+ in albustix, 0.3 g protein/L, 0.3 g protein/24 h, or 300 mmol protein/L.
Smuts et al., 2003 [13]	DHA-enriched eggs DHA 133 ± 15 mg median 7.3 eggs/week hence appr. 139 mg vs. simple eggs	291 (149 + 142) singleton pregnancies without any-cause hypertension	Mean 26.0 ± 1.4 weeks in main group and 26.1 ± 1.5 in control group Duration: until delivery	Criteria not listed since PIH and preeclampsia were secondary outcomes
Zhou et.al., 2012 [14] “DOMInO”	DHA 800 mg	2399 (1197 + 1202) singleton pregnancies, preeclampsia data was available for 96% cases	Median 19 (IQR 19–20) weeks in both groups Duration: until delivery	Hypertension: as either (1) one SBP reading of ≥160 mm Hg or DBP reading of ≥110 mm Hg or (2) 2 consecutive SBP readings of ≥140 mm Hg and/or DBP readings of ≥90 mm Hg 4 h apart after 20 wk gestation. Proteinuria as (1) a spot urine protein to creatinine ratio of ≥30 mg/mmol; (2) one 24-h urine specimen with a total protein content of ≥0.3 g/L on a dipstick test; or (3) a total protein content of ≥1 g/L from 2 random urine samples

* Data originally reported as means days. LA—linoleic acid, GLA—gamma-linolenic acid, EPA—eicosopentaenoic acid, DHA—docosohexaenoic acid, BP—blood pressure, SBP—systolic blood pressure, DBP—diastolic blood pressure, PIH—pregnancy-induced hypertension.
